# The effect of kV imaging dose on PTV and OAR planning constraints in lung SBRT using stereoscopic/monoscopic real‐time tumor‐monitoring system

**DOI:** 10.1002/acm2.70019

**Published:** 2025-02-21

**Authors:** Ruwan Abeywardhana, Mike Sattarivand

**Affiliations:** ^1^ Department of Medical Physics Nova Scotia Health Authority Halifax Nova Scotia Canada; ^2^ Faculty of Computer Science Dalhousie University Halifax Nova Scotia Canada; ^3^ Department of Physics & Atmospheric Science Dalhousie University Halifax Nova Scotia Canada; ^4^ Department of Radiation Oncology Dalhousie University Halifax Nova Scotia Canada

**Keywords:** image‐guided radiotherapy, imaging dose, organs at risk dose constraints, real‐time imaging, SBRT, tumor motion monitoring

## Abstract

**Purpose:**

Quantify the impact of additional imaging doses on clinical dose constraints during lung stereotactic body radiotherapy (SBRT) treatment utilizing stereoscopic/monoscopic real‐time tumor monitoring.

**Materials and Methods:**

Thirty lung SBRT patients treated with the volumetric arc therapy technique were randomly selected from the institutional clinical database. Contours of patients' and computed tomography data were extracted from the Eclipse treatment planning system, along with information regarding the treatment dose. Subsequently, patient‐specific three‐dimensional real‐time imaging dose distributions were computed using a validated Monte Carlo simulation of the ExacTrac imaging. The 3D imaging dose was added to the treatment dose, and the influence of the imaging dose on clinical dose constraints was analyzed for planning target volume (PTV) and various organs at risk (OARs).

**Results:**

Among the 30 patients, 14 patients exhibited one or more failed OAR constraints based solely on the treatment dose, resulting in a total of 24 constraint failures. The addition of the real‐time imaging dose altered the pass/fail criteria for one OAR constraint and two PTV constraints. The change in constraint due to additional imaging dose relative to the prescription dose was less than 1% for all patients, except for one case, where it reached 1.9%, which had remained below the threshold of 5% recommended by AAPM TG‐180 guidelines. Furthermore, the additional imaging dose relative to the treatment dose resulted in an increase in OAR constraints ranging from 0 to 27% (mean of 0.8%), with nine cases exceeding 5%.

**Conclusion:**

The current study represents the first attempt to investigate the impact of additional imaging doses on clinical planning constraints in real‐time tumor monitoring during lung SBRT utilizing ExacTrac imaging system. The addition of an imaging dose will likely have minimal clinical impact.

## 1 INTRODUCTION

Respiratory‐induced tumor motion introduces considerable geometrical and dosimetric uncertainties during thoracic radiation therapy.^1^ To mitigate these challenges, motion management techniques are employed to minimize planning target volume (PTV) margins during external beam radiation therapy for lung treatments. This is particularly important for patients undergoing stereotactic body radiation therapy (SBRT) of lung lesions where large therapeutic doses are delivered in a smaller number of fractions. In order to ensure accurate dose delivery and reduce PTV margin, various motion management techniques have been developed such as breath‐hold,^2^ gating,^3^ and tracking.^4^ These techniques often rely on imaging techniques during treatment planning and/or radiotherapy treatment sessions. Image‐guided radiation therapy (IGRT) has been established as a clinical standard for these patients and plays a crucial role in achieving geometric precision during lung SBRT,^5^, ^6^ as well as reducing PTV margins and optimizing dose delivery.^7^ To facilitate patient positioning and localize intrafraction lung tumor motion in real‐time,^8^ various tumor‐monitoring systems are utilized such as real‐time fluoroscopic kV imaging,^9^ Cyberknife (Accuray, Inc. Sunnyvale, CA),^10^ SyncTracX (Shimadzu Co., Kyoto, Japan),^11^ and ExacTrac (Brainlab AG, Germany).^12^ Most of these studies rely on room‐mounted projection x‐ray imaging systems and thus benefit from rapid image acquisition, high resolution, and minimal imaging dose^13^ compared to standard cone‐beam computed tomography (CBCT) suitable for pretreatment patient positioning.

Despite the significant reduction of PTV margins and optimization of dose delivery achieved by IGRT techniques, they may result in an additional imaging dose to critical organs at risk (OARs). Consequently, the quantification of imaging dose has been strongly advocated by the American Association of Physicists in Medicine Task Group 180 (AAPM TG‐180).^13^ Several studies have thus endeavored to quantify the three‐dimensional (3D) imaging dose distribution for thoracic regions for various image guidance systems, including CBCT,^14^, ^15^ on‐board kilovoltage (kV) and portal imaging,^16^ as well as real‐time fluoroscopic kV imaging,^17^ Cyberknife,^18^ SyncTraX system,^19^ and ExacTrac system.^20^

To mitigate the risk of radiation toxicity in OARs during lung SBRT, various fractionation criteria are employed during treatment planning,^21^, ^22^ and clinical dose constraints are defined. Depending on tumor size, location, and the number of fractions, OAR constraints may approach their tolerance limits with treatment dose alone. Consequently, delivering adequate prescription dose to cover PTV while ensuring adherence to acceptable OAR constraints often poses a challenge during treatment planning. Currently, during the treatment planning process, dose constraint calculations often ignore the additional imaging dose. However, real‐time imaging during treatment may significantly contribute to the radiation dose received by OARs particularly if they are close to their acceptable dose constraints. According to the AAPM‐TG‐180, the inclusion of imaging dose in the treatment planning process is only recommended if the imaging dose is above 5% of the prescription dose (Rx). However, no guidelines currently address the impact of imaging dose on planning parameters specifically on OAR clinical dose constraints. It is critical to investigate the effect of imaging dose on clinical dose constraints, mainly when OARs are near the PTV and are already receiving high treatment doses, thereby marginally meeting tolerance constraints.

To the best of the authors' knowledge, no prior study has examined the impact of imaging dose on clinical dose constraints. This study aims to quantify the influence of imaging dose derived from real‐time tumor monitoring utilizing stereoscopic/monoscopic real‐time kV imaging on PTV and OAR constraints. Here, we present findings from a retrospective analysis of 30 lung SBRT patients to assess the effect of real‐time imaging dose on PTV and OAR constraints.

## 2 MATERIALS AND METHODS

### 2.1 Patient selection and treatment planning

A total of 30 lung SBRT patients treated with volumetric arc therapy (VMAT) technique were randomly selected from Nova Scotia Health Cancer Center clinical database following a Research Ethics Board approval. Out of 30 patients, 26 patients were treated with a prescription of 48 Gy/4 fr, three with 60 Gy/8 fr, and one with 60 Gy/15 fr (Table 1). The fractionation was determined based on PTV location and the achievability of the predefined OAR constraints. All treatment plans were created by a qualified medical physicist and approved by a second independent physicist check then reviewed by a qualified radiation oncologist following the institutional lung SBRT clinical protocol using Eclipse treatment planning system version 15 (Varian Medical Systems, Palo Alto, CA). The OARs were lungs, trachea, esophagus, large bronchus (PB tree), heart, aorta, spinal cord, chest wall, and skin. A margin of 5 mm was added to trachea, esophagus, and spinal cord OARs to create the corresponding planning organ at risk volumes (PRV). Planning 4DCT was used to contour gross tumor volumes (GTV) at inhale and exhale phases as well as the maximum intensity projection (MIP) images. The three GTVs were then combined to create internal target volume (ITV) and a 5 mm margin was added to create the PTV.

**TABLE 1:** Patients' characteristics.

Prescription	numPt	Age (Std)	BMI (Std)	Gender (numPt)	Lobe (numPt)	Location (numPt)
48 Gy/4 fr	26	77.0 (±8.6)	27.6 (±6.8)	M (x9)	U (20)	Left (x10)
				F (x17)	M (3)	Right (x16)
					L (3)	
60 Gy/8 fr	3	82.6 (±4.0)	28.3 (1.3)	F (x3)	U (3)	Left (x1)
						Right (x2)
60 Gy/15 fr	1	80	41.7	M (x1)	U (1)	Right (x1)

Abbreviations: F, female; fr, number of treatment fractions; L, lower lobe; M, male; M, middle lobe; numPt, number of patients; Std, standard deviation; U, upper lobe.

### 2.2 Real‐time image guidance and imaging dose calculations

Patients’ pre‐treatment setup was performed using initial and verification stereoscopic image alignment followed by a CBCT verification. All the patients were treated without real‐time tumor monitoring as real‐time imaging in room‐mounted ExacTrac system is currently not enabled in clinical mode but possible in service mode. Real‐time imaging doses were calculated retrospectively assuming the patients went through real‐time stereoscopic / monoscopic image guidance using ExacTrac imaging system Ver 6.2 during their actual treatment fractions while the MV beam was on.^20^ The real‐time imaging frequency was 1.67 Hz, and the imaging techniques (kVp and mAs) were patient‐size‐dependent and were based on vendor recommended values. Although ExacTrac is a stereoscopic room‐mounted imaging system, during a realistic treatment fraction delivery throughout a VMAT arc, one of the x‐ray views would be periodically blocked by the rotating linac gantry^23^, ^24^ (see fig. 1 in Ref.^23^). Therefore, real‐time imaging was divided into stereoscopic and monoscopic periods based on the gantry angle for each VMAT arc to calculate realistic imaging dose. For stereoscopic periods, imaging dose was calculated from both views while for monoscopic periods imaging dose was calculated from only the nonblocked view.

Patient's contours and CT data were exported from the Eclipse treatment planning system along with the treatment dose distribution. The patient‐specific 3D imaging dose distribution was calculated using a validated Monte‐Carlo (MC) simulation of the ExacTrac imaging system modeling stereoscopic / monoscopic real‐time imaging using DOSEXYZnrc.^25^ The 3D imaging dose data in the current study shares the same data for 30 lung patients from Abeywardhana et al.^20^ Briefly, it models the geometry of the ExacTrac imaging system using EGSnrc code,^26^, ^27^ and BEAMnrs^28^ was utilized to model the ExacTrac tube and generate phase space files for different x‐ray energies.^25^ The 3D dose distribution of a sample lung patient, in coronal, sagittal, and axial views is presented in (fig. 3 in Ref.^20^).

### 2.3 Plan metrics evaluation using treatment and imaging dose

The calculated 3D real‐time imaging doses were added to the treatment dose, and the effect of the imaging dose on constraints was analyzed for PTV and OARs using an in‐house Matlab software (MathWorks, Natick, MA). The constraints were analyzed for treatment dose (Tx) and total dose (Tx + Im), and the constraints changes due to imaging dose (Im) were calculated. The constraints for 4, 8, and 15 fractions were based on RTOG 0915,^29^ LUSTER,^30^ SUNSET^31^ clinical trials respectively (Table 2). The OAR constraints were calculated based on the dose‐volume histogram (DVH). To enhance comparability, the constraints in Table 2 were studied in three categories: absolute dose constraints (C1), absolute volume constraints (C2), and volume percentage constraints (C3). C1 was based on the average dose (Dmean), maximum dose (Dmax), or dose at 2 percent volume (D2%) of an OAR in units of (Gy). Dmax was defined as the dose received by 0.03 cc volume of the OAR.^32^, ^33^ The absolute volume constraints (C2) were calculated in units (cc). For instance, Trachea PRV (V15.6  Gy) was the volume of Trachea PRV in cc that received a radiation dose higher than 15.6 Gy. The volume percentage constraints (C3) were the constraints defined as a volume percentage of the OAR or PTV, e.g., Lt‐Lung V20 Gy was the volume percentage of the left lung receiving a dose higher than 20 Gy. Even though bones are typically not listed among the clinical OAR constraints, this study specifically incorporates bone along with the other OARs due to its ability to receive much higher kV imaging dose compared to soft tissue. It is noted that the chest wall alone may not adequately encompass all bone volume receiving high imaging dose, particularly considering that the ExacTrac kV imaging beams are predominantly posterior thus monitoring posterior lung tumors might add significant imaging dose to posterior bones. Thus, in addition to the clinical OAR constraints, bone D2%^19^, ^20^ and V30 Gy (analogous to ChestWall) were calculated in the current study.

**TABLE 2:** PTV and OAR constraints for all different fractionations.

Category	Constraint	48 Gy/4 fr (RTOG 0915)	60 GY/8 fr (LUSTER)	60 Gy/15 fr (SUNSET)
C3	PTV V90%	>99%	>99%	>99%
C3	Lt Lung V20Gy	≤10%	≤10%	≤10%
C1	Lt Lung Dmean	≤6 Gy	≤6 Gy	≤14 Gy
C3	Rt Lung V20Gy	≤10%	≤10%	≤10%
C1	Rt Lung Dmean	≤6 Gy	≤6 Gy	≤14 Gy
C2	Trachea PRV	V15.6 Gy ≤ 4cc	V60 Gy ≤ 5cc	V62 Gy ≤ 10cc
C1	Trachea PRV Dmax	≤34.8 Gy	≤64 Gy	≤66 Gy
C2	Esophagus PRV	V18.8 Gy ≤ 5 cc	V22 Gy ≤ 5 cc	V48 Gy ≤ 5 cc
C1	Esophagus PRV Dmax	≤30 Gy	≤40 Gy	≤55.5 Gy
C2	PB Tree	V15.6 Gy ≤ 4 cc	V60 Gy ≤ 5 cc	V62 Gy ≤ 10 cc
C1	PB Tree Dmax	≤34.8 Gy	≤64 Gy	≤66 Gy
C2	Heart	V28 Gy ≤ 15 cc	V60 Gy ≤ 10 cc	V62 Gy ≤ 10 cc
C1	Heart Dmax	≤34 Gy	≤64 Gy	≤66 Gy
C2	Aorta	V28 Gy ≤ 15 cc	V60 Gy ≤ 10 cc	V60 Gy ≤ 10 cc
C1	Aorta Dmax	≤ 34 Gy	≤64 Gy	≤ 64 Gy
C2	Spinal Cord PRV	V20.8 Gy ≤ 0.35 cc	V22 Gy ≤ 1 cc	N/A
C2	Spinal Cord PRV	V13.6 Gy ≤ 1.2 cc	N/A	N/A
C1	Spinal Cord PRV Dmax	≤26 Gy	≤32 Gy	≤42 Gy
C2	ChestWall	V30 Gy ≤ 70 cc	V30 Gy ≤ 70 cc	V30 Gy ≤ 70 cc
C2	Skin	V33.2 Gy ≤ 10 cc	V40 Gy ≤ 10 cc	N/A
C1	Skin Dmax	<36 Gy	<45 Gy	N/A
C2	Bone V30 Gy [cc]^a^	N/A	N/A	N/A
C1	Bone D2% [Gy]^a^	N/A	N/A	N/A

Abbreviations: fr, number of treatment fractions; Lt, left; Rt, right.

^a^ Bone metrics are not from the clinical protocols but are included for comparison purposes.

## 3 RESULTS

**FIGURE 1:**
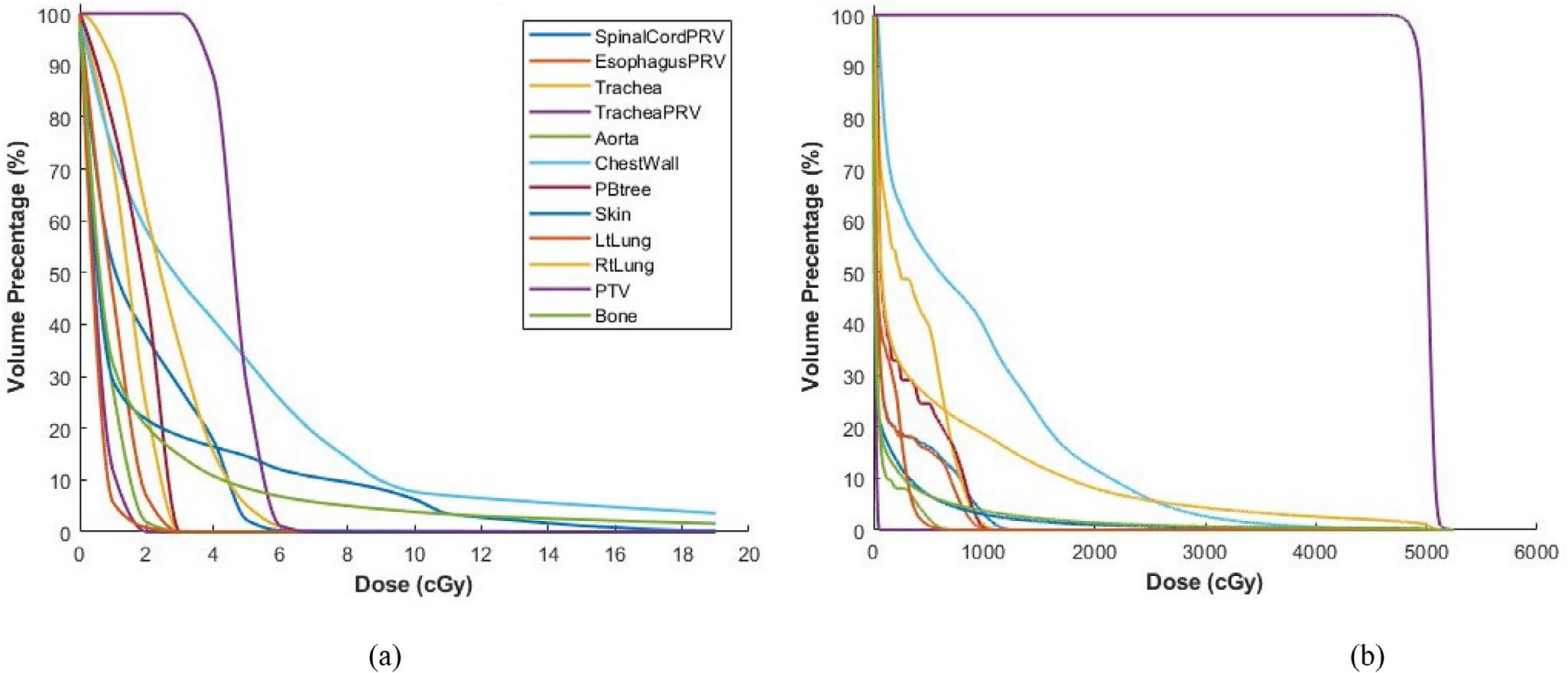
DVHs for (a) imaging dose and (b) treatment dose.

Figure 1 illustrates the DVHs from imaging dose (Figure 1a) and treatment dose (Figure 1b) for PTV and OARs of a sample patient with a 48 Gy/4 fr prescription.

Table 3 presents the PTV and OAR constraints before and after including the imaging dose for the patient who was prescribed with 60 Gy/15 fr. The last two columns indicate the increase in constraint value due to imaging relative to treatment and prescription, that is, Im/Tx (%) and Im/Rx (%), respectively. The last column applies only to C1 category constraints. For this patient, all the constraints were met by the Tx dose, and the additional imaging dose did not cause any failure of the constraints. In this case, the imaging dose relative to treatment or prescription was both negligible.

**TABLE 3:** PTV and OAR constraints for the 60 Gy/15 fr patient (SUNSET).

	Constraint	Requirement	Tx	Tx + Im	Im/Tx (%)	Im/Rx (%)
C3	PTV V90%	>99%	99.1	99.1	0.04	–
C3	Lt Lung V20 Gy	≤10%	0.0	0.0	0.00	–
C1	Lt Lung Dmean	≤14 Gy	3.3	3.3	0.10	0.01
C3	Rt Lung V20 Gy	≤10%	**30.4**	**30.4**	0.04	–
C1	Rt Lung Dmean	≤14 Gy	**16.7**	**16.7**	0.08	0.02
C2	Trachea PRV V62 Gy	≤10cc	0.0	0.0	0.00	–
C1	Trachea PRV Dmax	≤66 Gy	23.6	23.6	0.08	0.03
C2	EsophagusPRV V48 Gy	≤5 cc	0.0	0.0	0.00	–
C1	EsophagusPRV Dmax	≤50.5 Gy	24.5	24.5	0.08	0.03
C2	PB Tree V62Gy	≤10 cc	0.0	0.0	0.00	–
C1	PB Tree Dmax	≤ 66 Gy	21.8	21.8	0.09	0.03
C2	Heart V62Gy	≤10 cc	0.0	0.0	0.00	–
C1	Heart Dmax	≤66 Gy	4.9	4.9	0.24	0.02
C2	Aorta V60Gy	≤10 cc	0.0	0.0	0.00	–
C1	Aorta Dmax	≤64 Gy	13.1	13.1	0.08	0.02
C1	SpinalCordPRV Dmax	≤42 Gy	21.0	21.0	0.06	0.07
C2	ChestWall V30Gy	<70 cc	23.9	23.9	0.16	
C1	Skin Dmax	N/A	64.8	64.8	0.07	0.00
C2	Bone V30Gy	N/A	21.1	21.1	0.25	
C1	Bone D2%	N/A	36.1	36.2	0.14	0.01

*Note*: Values in bold indicate unmet constraints. Column labels: Im/Tx (%) = Im dose as a percentage of Tx dose, Im/Rx (%) = Im dose as a percentage of Rx dose.

Table 4 presents the PTV and OAR constraints of the three patients who were prescribed 60 Gy/8 fr. As before, the imaging dose (Im) is presented as a percentage of the Tx dose and also as a percentage of Rx in the last two columns for each patient. The original treatment plan of the first patient did not fulfill the PTV V90% constraint (values in **bold**) and this did not change after adding the imaging dose. Similarly, constraints of the left lung in Patient 2 and right lung in Patient 3 were unmet both before and after the addition of the imaging dose. Although the Im/Rx(%) values were negligible for these three patients, the highest Im/Tx (%) value was 1.9%, which was observed for the first and the third patients’ heart Dmax constraint.

**TABLE 4:** PTV and OAR constraints for the 60 Gy/8 fr patients (LUSTER).

			Patient 1				Patient 2				Patient 3			
Category	Constraint	Requirements	Tx	Tx + Im	Im/Tx (%)	Im/Rx (%)	Tx	Tx + Im	Im/Tx (%)	Im/Rx (%)	Tx	Tx + Im	Im/Tx (%)	Im/Rx (%)
C3	PTV V90%	>99%	**98.7**	**98.7**	0.06	–	99.4	99.4	0.02	–	99.0	99.1	0.06	–
C3	Lt Lung V20 Gy	≤10%	4.9	4.9	0.16	–	**13.9**	**14.0**	0.15	–	0.0	0.0	0.00	–
C1	Lt Lung Dmean	≤6 Gy	3.7	3.7	0.36	0.02	**7.8**	**7.8**	0.28	0.04	1.5	1.5	0.62	0.02
C3	Rt Lung V20 Gy	≤10%	0.0	0.0	0.00	–	0.0	0.0	0.00	–	**13.4**	**13.4**	0.20	–
C1	Rt Lung Dmean	≤6 Gy	1.3	1.3	0.52	0.01	1.8	1.8	0.38	0.01	**6.9**	**6.9**	0.33	0.04
C2	Trachea PRV 60 Gy	≤5 cc	0.0	0.0	0.00	–	0.0	0.0	0.0	–	0.0	0.0	0.00	–
C1	Trachea PRV Dmax	≤64 Gy	10.6	10.6	0.25	0.04	17.1	17.1	0.19	0.05	25.9	26.9	0.13	0.06
C2	Esophagus PRV V22 Gy	≤5 cc	0.0	0.0	0.00	–	0.0	0.0	0.00	–	2.2	2.2	0.53	–
C1	Esophagus PRV Dmax	≤40 Gy	11.9	11.9	0.23	0.05	16.9	16.9	0.13	0.04	34.4	34.4	0.09	0.05
C2	PB Tree V60 Gy	≤5 cc	0.0	0.0	0.00	–	0.0	0.0	0.00	–	0.0	0.0	0.00	–
C1	PB Tree Dmax	≤64 Gy	23.0	23.0	0.14	0.05	32.2	32.3	0.07	0.04	16.9	16.9	0.18	0.05
C2	Heart V60 Gy	≤10 cc	0.0	0.0	0.00	–	0.0	0.0	0.00	–	0.0	0.0	0.00	–
C1	Heart Dmax	≤ 64 Gy	0.1	0.1	1.86	0.00	3.4	3.4	0.52	0.03	1.2	1.2	1.91	0.04
C2	Aorta V60 Gy	≤10 cc	0.0	0.0	0.00	–	0.0	0.0	0.00	–	0.0	0.0	0.00	–
C1	Aorta Dmax	≤64 Gy	19.6	19.6	0.14	0.04	32.5	32.5	0.12	0.06	21.7	21.7	0.33	0.12
C2	Spinal Cord PRV V22 Gy	≤1 cc	0.0	0.0	0.00	–	0.0	0.0	0.00	–	0.0	0.0	0.00	–
C1	Spinal Cord PRV Dmax	≤32 Gy	9.4	9.4	0.23	0.11	14.8	14.8	0.14	0.23	18.3	18.4	0.16	0.17
C2	ChestWall V30Gy	≤70 cc	4.5	4.6	1.43	–	51.2	52.3	0.27	–	18.1	18.2	0.58	–
C2	Skin V40Gy	≤10 cc	0.0	0.0	0.00	–	0.0	0.0	0.00	–	0.0	0.0	0.00	–
C1	Skin Dmax	≤45 Gy	20.6	20.6	0.28	0.15	26.9	27.0	0.04	0.17	28.8	28.9	0.42	0.20
C2	Bone V30 Gy [cc]	N/A	63.7	63.8	0.14	–	47.0	47.1	0.21	–	40.4	40.6	0.30	–
C1	Bone D2% [Gy]	N/A	22.0	22.1	0.17	0.04	22.0	22.0	0.21	0.03	15.9	16.0	0.45	0.08

*Note*: Values in **bold** indicate unmet constraints. Column names: Im/Tx (%) = Im dose as a percentage of Tx dose, Im/Rx (%) Im dose as a percentage of Rx dose.

**FIGURE 2:**
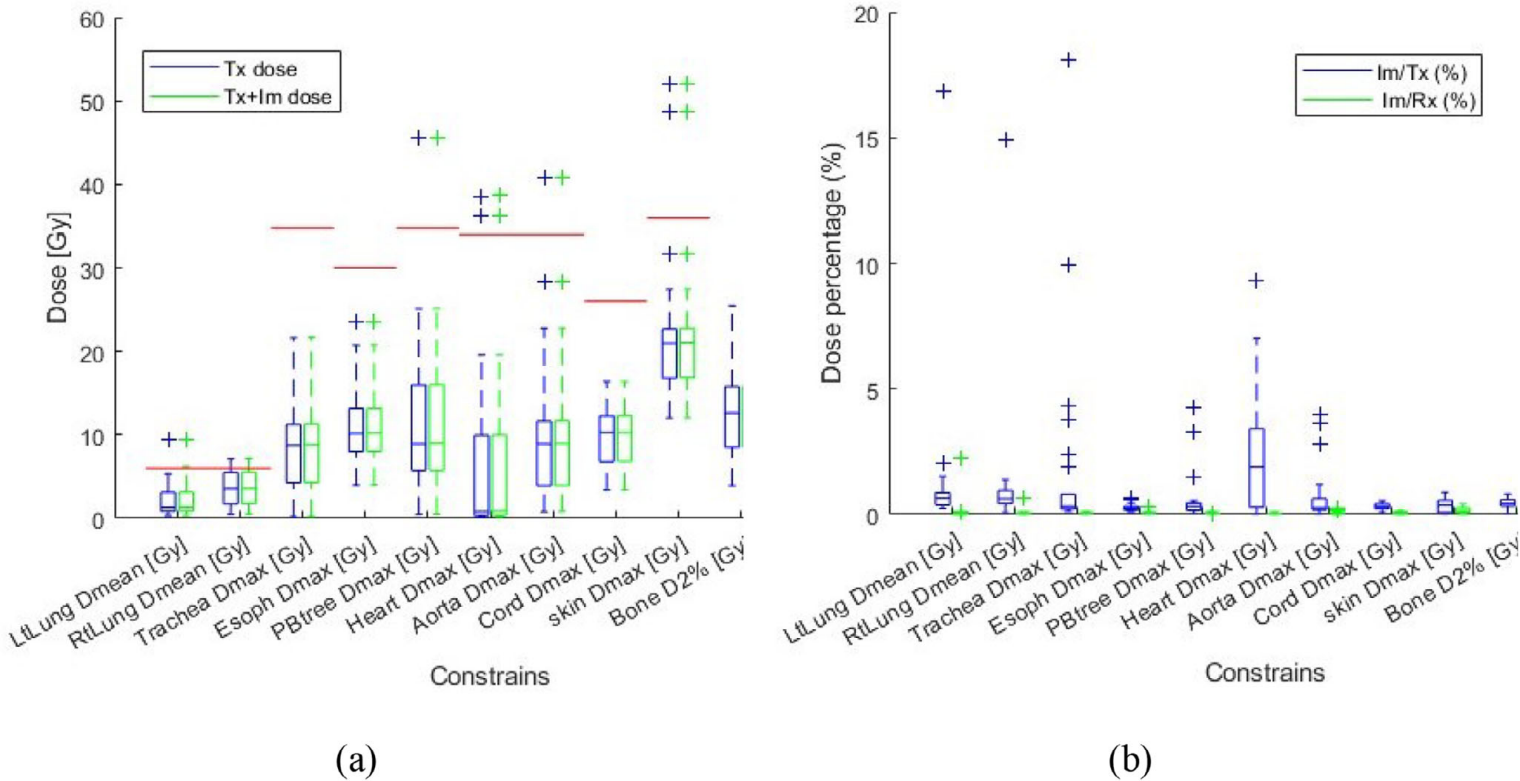
Category 1 (C1) OAR dose constraints for 48 Gy/4 fr patients. (a) Tx dose (blue) and Tx + Im dose (green). The red horizontal lines represent the constraints in Table 2. (b) Im doses relative to the Tx dose (blue) and relative to the Rx dose (green).

**FIGURE 3:**
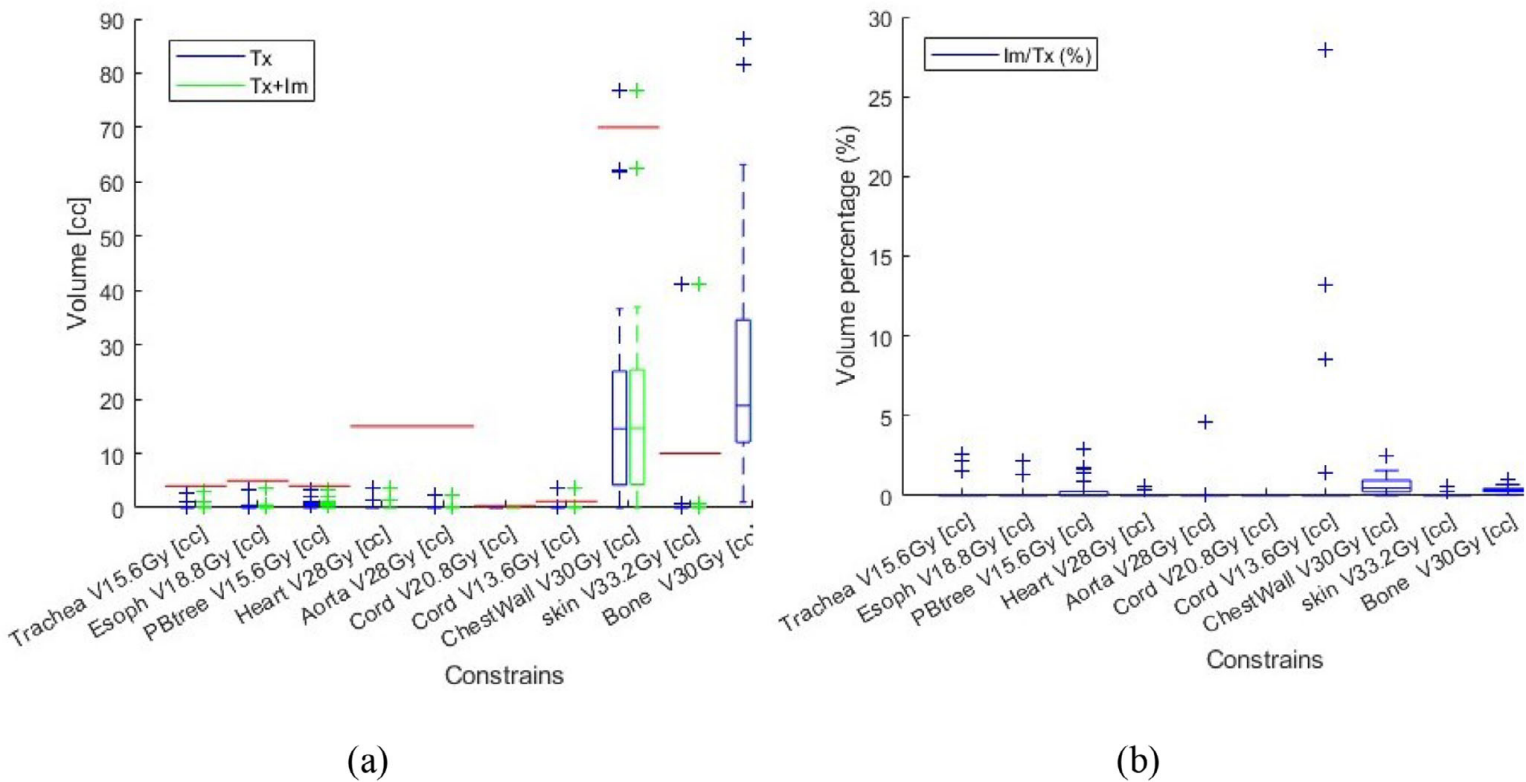
Category 2 (C2) OAR dose–volume constraints for 48 Gy/4 fr patients. (a) Volumes receiving Tx dose alone (blue) volumes receiving Tx + Im dose (green). The red horizontal lines represent the OAR constraints as per Table 2. (b) OAR volume constraint increases due to imaging dose as a percentage of OAR volume constraint from Tx dose.

**FIGURE 4:**
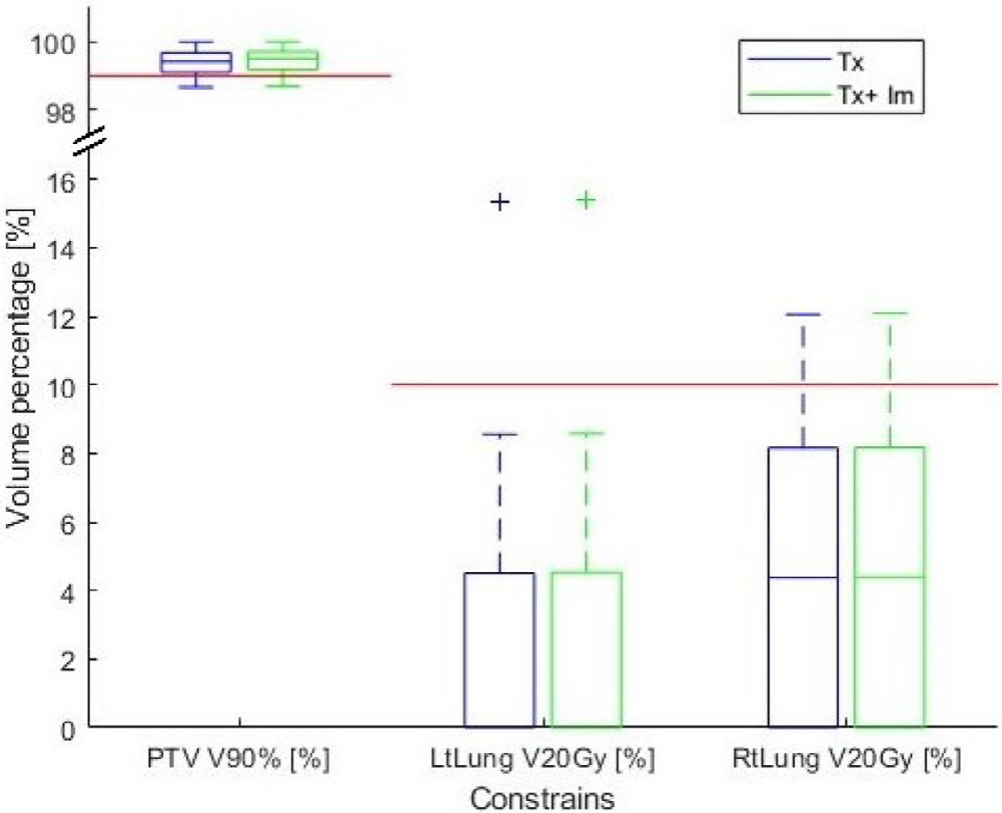
Category 3 (C3) PTV and OAR dose–volume constraints for 48 Gy/4 fr patients. Volume percentages from the Tx dose (blue) and volume percentages from the Tx + Im dose (green). The red horizontal lines represent the constraints for volume percentage values as per Table 2.

Figures 2, 3, 4 illustrate the PTV and OAR constraints before and after the addition of imaging dose for the 26 patients prescribed with 48 Gy/4fr. Each figure illustrates one of the three categories of constraints, that is, C1 (Figure 2), C2 (Figure 3), and C3 (Figure 4). Figure 2b illustrates dose constraint data in Figure 2a but indicates its increase relative to treatment dose or prescription dose, Im/Tx (%) and Im/Rx (%), respectively. Similarly, Figure 3b demonstrates volume constraint data in Figure 3a but indicates its increase relative to treatment, Im/Tx (%). The red horizontal lines in the figures indicate the clinical constraints for each OAR or PTV as per Table 2.

Out of all 30 patients, 14 patients had one or more failed OAR constraints with treatment dose alone. With a total of 535 OAR constraints across 30 lung SBRT patients, 24 constraints failed to meet the acceptable limit (4.5% out of 535) with Tx dose alone (above the red horizontal lines in Figures 1, 2, and 3, or bold in Tables 3 and 4). The real‐time imaging dose has effectively changed the pass/fail criteria of only one OAR constraint out of a total of 535 (0.2% of cases). In this case, the left lung Dmean [< 6 Gy] constraints were met in the original treatment plane with a Dmean of 5.3 Gy. However, after adding the imaging dose, the left lung Dmean increased to 6.2 Gy, failing the 6‐Gy constraint (Figure 2a). The paired *T*‐test showed that the BoneV30 Gy [cc] constraint value was higher than that of the chest wall (*p* < 0.05) with average values of 28.0 versus 19.6 cc for bone and chest wall, respectively (Figure 3a).

Out of all the 30 PTV constraints, eight constraints were unable to meet the acceptable limit with the Tx dose alone; however, after adding the imaging dose, the number of failed PTV constraints was reduced to 6. Two of the PTV V90% [>99%] constraints failed in the original treatment plan with a PTV volume percentage of 98.96 and 98.98%, respectively. However, after the additional imaging dose, the PTV V90% constraint was met (Figure 4) with volume percentages increasing to 99.10 and 99.07%, respectively. For both cases, the PTV constraints changed from marginal fails to marginal passes altering the pass/fail condition.

Additional imaging dose relative to Rx for all patients’ OARs was less than 1%, except in one case, it was 1.9% (Figure 1b), which was still less than the 5% recommended value in TG‐180. The imaging dose relative to the Tx dose increased the OAR constraints in the range [0%–27%] (mean 0.8%, nine cases > 5%) (Figures 1b and 2b).

## 4 DISCUSSION

This study quantifies the patient‐specific real‐time imaging dose from real‐time stereoscopic/monoscopic image guidance system and its impact on clinical dose constraints in 30 patients undergoing lung SBRT with various fractionations (48 Gy/4 fr, 60 Gy/8 fr, and 60 Gy/15 fr). The results showed that the additional imaging dose applied to the PTV and OARs constraints remained relatively small compared to the prescription dose, with values consistently below 1%, except for one case where it reached 1.9%. However, the effect of the additional imaging dose on the constraints, compared to the dose received by the Tx dose, exhibited higher percentages in certain instances, ranging from 0 to 27% (mean of 0.8%).

The real‐time imaging dose can potentially influence the pass/fail criteria of the PTV and/or OAR constraints. We have observed two instances where a PTV V90% (>99%) constraint marginally failed based on the treatment dose. However, these constraints were marginally passed after considering the additional real‐time imaging dose. Conversely, in one case, the left lung Dmean [Gy] constraint initially passed based on the Tx dose while it failed upon the addition of the imaging dose.

Out of all the clinical constraints in the C1 category, the skin Dmax showed the highest Tx dose (Figure 2a), where the constraint was also a higher value. Unlike the treatment dose with the MV beam, the maximum dose with kV beams occurs at the surface. A prior study^20^ demonstrated that the real‐time imaging dose from kV stereoscopic/monoscopic imaging could deliver a D2 skin up to 0.95 Gy, that is, 2.0% of their Rx dose. Here, D2 represents the dose received by 2% of the OAR volume, a value lower than the Dmax. However, due to ExacTrac's oblique beam geometry, the irradiated skin by the kV beams is often not overlapped by the skin irradiated by the coplanar MV treatment beams. Thus, there is only a very small chance of the volume of treatment Dmax overlapping with imaging Dmax except for very posterior tumors. For example, in the current study, the maximum increment of skin Dmax constraints values caused by additional real‐time imaging dose was only 0.35% of the Rx dose (Figure 2b), and it did not alter the pass/fail criteria.

Since the PTV location within the lung varies significantly among the patients, the imaging dose received by an OAR from stereoscopic/monoscopic exhibits a high standard deviation.^20^ Given that the stereoscopic/monoscopic imaging dose is primarily directed posteriorly, the impact of the imaging dose on posterior OARs, such as the spinal cord, could be more noticeable. We have observed the highest Im/Tx (%) value of 27% in the Cord V13.6  Gy constraint (Figure 3b). In this case, the constraint for Cord V13.6  Gy had received a very low Tx dose (1.2% of OAR constraint).

Although bone does not explicitly have a clinical constraint in lung SBRT protocols, dose metrics for bone were included in the current study since the bone dose absorption coefficient is ∼4 times higher for kilovolt than that for megavolt.^34^ We have observed that for the constraints in the C2 category, bone has a higher value (Figure 3a). Furthermore, a previous study^20^ has reported that real‐time imaging with the ExacTrac system could deliver a D2 up to 2.06 Gy to bones, which is 4.3% of the Rx dose. Given that the clinical constraint for the chest wall was based on the volume receiving 30 Gy (V30 Gy), a similar metric was utilized for bone, that is, V30 Gy. The result indicates that bones received a statistically higher treatment dose than the chest wall. This can be explained by the fact that chest wall volume includes both bone and soft tissue.

Since the 3D dose distributions of the treatment dose and imaging dose differ dramatically, the location of the maximum treatment dose within a particular OAR does not coincide with that of the maximum imaging dose. As an example, skin Dmax change due to imaging dose (different between Tx and Tx + Im) is not the same as the imaging dose Dmax that is calculated based on imaging does DVH alone. Consequently, even if the maximum imaging dose constitutes a significant percentage of the treatment dose, it does not substantially alter the combined dose from treatment and imaging for Dmax constraints.

There are number of studies in the literature that report imaging doses for various technologies and treatment sites. Table 5 shows a comparison of studies that quantified the imaging dose from real‐time tumor monitoring. Shirato et al.^35^ and Depuydt et al.^36^ have measured the surface dose delivered from and RTRT and Brainlab Vero system to be 98.0 cGy/h and 1.76–3.52 cGy, respectively. Juneja et al.^9^ and Legge et al.^37^ have reported surface dose and absorbed dose by PTV from fluoroscopic imaging dose with fiducial marker utilizing Kilovoltage Intrafraction Monitoring (KIM) technology during thoracic IGRT (skin does = 0.5–2.6 cGy) and Pelvic IGRT (skin does = 2.74 ± 0.19 cGy), respectively. Similarly, Spezi et al.^15^ and Ding et al.^16^ have utilized MC‐EGSnrc technique to calculate the mean organ dose (<6 cGy) from Elekta XVI and organ D50 (heart = 0.42 cGy; bladder = 1.4 cGy) from VarianOBI, respectively. Moeckli et al.^18^ have calculated the effective dose from the CyberKnife system, and reported maximum effective dose was 4.2 cSv per fraction for a lung patient. Shiinoki et al.^19^ and Abeywardhana et al.^20^ have reported D2 and D50 values for PTV and OARs during real‐time imaging dose using SyncTraX system (skin D2 = 2.65–93.58 cGy) and ExacTrac system (skin D2 = 52.2 ± 21.4 cGy), respectively. Unlike previous literature that reported selected dose parameters from imaging dose (Im) alone for the OARs, the current study reports the dose/volume parameters based on clinical dose–volume constraints used during the actual treatment planning process (Table 2) based on total does (Rt + Im). This is critical since only the effect of the imaging dose on planning PTV and OAR constraints will directly determine its importance in clinical settings. Moreover, some constraints are volume constraints, and some are percentage of volumes rather than dose constraints (e.g., V60 Gy [cc] or V20 Gy [%] in Table 2) which are not previously reported. For these reasons, a direct comparison of the results from this study (planning constraints from total dose) and previous studies (selected imaging dose parameters) is not feasible (Table 5).

**TABLE 5:** Summary of real‐time kV imaging dose studies with different tumor‐monitoring technologies and the reported parameters.

Author	Technology	Method	Region/Site	numPt	Parameter
Shirato et al.^35^	Mitsubishi RTRT	Measurement	Phantom	1	Surface dose
Depuydt et al.^36^	Vero 4DRT	Measurement	Lung, liver	10	Surface dose
Juneja et al.^9^	KIM	Measurement	Lung	10	Point dose measurement
Legge et al.^37^	KIM	Measurement	Prostate	1	Point dose measurement
Spezi et al.^15^	Elekta XVI	MC‐EGSnrc	Lung, prostate, head	6	Mean dose (PTV)
Ding and Munro (2013)^16^	Varian OBI	MC‐EGSnrc	Lung, prostate, head	3	D50 OARs
Moeckli et al.^18^	CyberKnife	MC‐GEANT4	Lung, prostate, head	20	Effective dose
Shiinoki et al.^19^	SyncTraX	MC‐EGSnrc	Lung	10	D2 OARs
Abeywardhana et al.^20^	ExacTrac	MC‐EGSnrc	Lung, prostate	60	D50, D2 OARs
Current study	ExacTrac	MC‐EGSnrc	Lung	30	PTV and OAR clinical treatment planning constraints

Abbreviation: numPt, number of patients.

AAPM TG‐180 guidelines recommend maintaining an imaging dose lower than 5% of the Rx dose to avoid its inclusion in the planning process. This recommendation can only be applied to the C1 category which is dose‐based constraints since C2 and C3 constraints are measured in units of volume and percentage. No recommendations are currently available for the effect of imaging dose on OAR clinical constraints. Thus, consensus recommendations are required for OAR constraints especially those that are other than dose‐based constraints. In the current study, the highest observed OAR imaging dose in the C1 category was 1.9% of Rx for the left lung Dmean [Gy]. Overall observation has provided evidence that even if an OAR receives a high imaging dose as a percentage of Rx, its effect on altering the constraint value due to additional imaging dose is much smaller since the 3D dose distributions between treatment and imaging are vastly different and the constraint values are largely affected by treatment dose, which is orders of magnitude larger than the imaging dose (Figure 1).

To facilitate target localization during kV real‐time tumor monitoring, implanted fiducials may be used as reported for image guidance in the prostate using the ExacTrac system.^24^ However, for lung tumors, the implanting fiducial carries a considerable risk of pneumothorax.^38^, ^39^ Thus, several studies^40^ reported on markerless lung tumor tracking for various kV image‐guided systems such as ExacTrac,^12^ Vero 4DRT,^41^, ^42^ CyberKnife,^43^ and linac‐mounted kV imaging.^44^, ^45^, ^46^, ^47^

For pretreatment patient positioning in IGRT utilizing ExacTrac imaging system, two initial and verification CBCT scans were acquired for lung patients as per our treatment protocol. These pretreatment CBCT scans contribute additional imaging doses to the OARs. Numerous studies have been conducted to quantify the organ‐specific imaging dose from CBCT scans.^16^, ^48^, ^49^ According to a previous study,^14^ the dose received by 50% of the organ volume (D50) from a single CBCT scan ranges from 3.0 to 7.1 mGy. The dose delivered during the two initial and verification CBCT scans is less than 1% of the ExacTrac real‐time imaging dose.^20^ Moreover, the PTV margins used here are based on a standard pretreatment imaging protocol without real‐time tumor monitoring. However, when patients undergo treatment with real‐time tumor monitoring, the ITV margin can be minimized to only residual motion. This could lead to a further reduction in the total treatment and imaging dose to OARs,^50^ an effect which is not considered in the current study. This suggests that adding real‐time imaging dose to track tumor motion in real‐time may not be a limiting factor for real‐time tumor tracking.

To the authors' knowledge, the current study represents the first attempt to investigate the impact of imaging dose from the ExacTrac imaging system on clinical OAR constraints during lung SBRT. Shiinoki et al. have compared imaging dose estimation for different imaging techniques (tab. 5 in Ref.^19^) and showed the real‐time imaging dose over the course of treatment is in the same range despite differences in the imaging parameters (e.g., kV, mAs). Similarly, a previous study^20^ showed the real‐time imaging dose from ExacTrac system is in the same order of magnitude compared to previous real‐time imaging studies with other IGRT techniques (tab. 4 in Ref.^20^). This suggests that the result of the current may be generalizable to other real‐time imaging technologies, particularly considering that the 3D dose distribution between imaging and treatment is vastly different and the OAR constraints are mainly affected by treatment dose.

The current study is focused on investigating real‐time tumor monitoring in lung‐SBRT. Compared to other treatment sites, lung tumors exhibit the largest motions of up to 3 cm during a regular breathing cycle,^8^, ^51^, ^52^ which may require large marginal uncertainty.^53^ Similarly, other treatment sites (e.g., prostate) may reveal similar results in terms of the effect of imaging dose on OAR constraints. For instance, Abeywardhana et al. showed that the additional real‐time imaging dose during prostate SBRT is lower compared to lung SBRT real‐time tumor monitoring (figs. 4 and 6 in Ref.^20^). For a more comprehensive understanding of the influence of imaging dose on OAR constraints, further research is warranted across different treatment sites and utilizing various tumor‐monitoring imaging techniques.

## 5 CONCLUSION

This study assessed patient‐specific imaging doses for real‐time kV stereoscopic/monoscopic image guidance in 30 lung SBRT patients and quantified its impact on PTV and OAR constraints. Among 535 OAR constraints and 30 PTV constraints, only one (0.2%) constraint changed from pass to fail by additional imaging dose, with imaging dose representing 1.9% of the prescription dose. All OAR constraint increments due to the imaging dose were within the recommended <5% value by the AAPM TG‐180. Overall, the findings suggest that the imaging dose from the stereoscopic/monoscopic real‐time image guidance did not critically affect the pass/fail criteria of OAR constraints.

## AUTHOR CONTRIBUTIONS

*Data analysis and interpretation; Manuscript writing*: Ruwan Abeywardhana. *Conceptualization, Collection and assembly of data, Manuscript writing, Review and editing of the work: Mike Sattarivand. All authors have read the published version of the manuscript and agreed on all aspects of the work*.

## CONFLICT OF INTEREST STATEMENT

The authors declare no conflicts of interest.
